# Supplementary material for: Case-area targeted preventive interventions to interrupt cholera transmission: Current implementation practices and lessons learned
Source: PLoS Negl Trop Dis. 2021 Dec 17;15(12):e0010042. doi: 10.1371/journal.pntd.0010042 (PMC8719662; doi:10.1371/journal.pntd.0010042)
Supplement: S1 Table — (DOCX) [file pntd.0010042.s001.docx]

**S1 Table: Literature Search Strategy Summary**

| Peer Reviewed Sources | |
| --- | --- |
| PubMed, EMBASE | Concept 1: Cholera, cholera outbreak, cholera disease outbreak, cholera epidemic, cholera prevention, or cholera control  AND  Concept 2: Rapid response team, surge capacity, medical emergency team, emergency response, intervention, or case-area targeted intervention |
| **Grey Literature Sources** | |
| Google (manual search, advanced search and google scholar) | Cholera epidemic, cholera outbreak, cholera intervention, cholera response, cholera rapid response team, cholera emergency response, cholera surge capacity |
| Relief Web | Keyword search for cholera and cholera response within updates and reports (disaster type=outbreaks) |
| Technical and/or Coordinating Bodies | Assessment Capacities Project (ACAPs); Cholera Platform; Centers for Disease Control; the German WASH Network; the Global Alliance against Cholera (GAC); the Global Emergency Response Coalition; the New Humanitarian; Sustainable Water and Sanitation Alliance; Sphere; UNICEF; World Health Organization (WHO); and World Water Week |
| Implementing Organizations | Action Against Hunger (ACF - France and US); Arche Nova; Concern; GOAL; ICDDR, B; International Medical Corps (IMC); International Rescue Committee (IRC); Medair; Médecins sans Frontiers (MSF); Oxfam; Red Cross/Red Crescent Societies (IFRC and ICRC); Save the Children; and Solidarités International (SI) |
| Donor Organizations | US Agency for International Development, Bureau for Humanitarian Assistance (BHA) |
