# Supplementary material for: Case-area targeted preventive interventions to interrupt cholera transmission: Current implementation practices and lessons learned
Source: PLoS Negl Trop Dis. 2021 Dec 17;15(12):e0010042. doi: 10.1371/journal.pntd.0010042 (PMC8719662; doi:10.1371/journal.pntd.0010042)
Supplement: Attachment — Submitted filename: Reviewer report _ Manuscript PNTD-D-21-00779_ Godfrey Bwire.pdf [file pntd.0010042.s002.pdf]

Reviewer Recommendation and Comments for Manuscript Number PNTD-D-21-00790

Challenges, Best Practices and Lessons Learned from the first Mass vaccination campaign in Urban Settings of Cameroon in the COVID-19 era.

Original Submission
Godfrey Bwire, MB Ch B, MPH

Back | Edit Review | Print | Submit Review to Editorial Office

Recommendation: Reject

**Custom Review Question(s):**	**Response**
**Key Review Criteria Required for Acceptance?**<br><br>As you describe the new analyses required for acceptance, please consider the following:<br><br>**Methods**<br><br>-Are the objectives of the study clearly articulated with a clear testable hypothesis stated?<br>-Is the study design appropriate to address the stated objectives?<br>-Is the population clearly described and appropriate for the hypothesis being tested?<br>-Is the sample size sufficient to ensure adequate power to address the hypothesis being tested?<br>-Were correct statistical analysis used to support conclusions?<br>-Are there concerns about ethical or regulatory requirements being met?	Are the objectives of the study clearly articulated with a clear testable hypothesis stated? (Yes)<br>-Is the study design appropriate to address the stated objectives? (NO)<br>-Is the population clearly described and appropriate for the hypothesis being tested? (YES)<br>-Is the sample size sufficient to ensure adequate power to address the hypothesis being tested? (NA)<br>-Were correct statistical analysis used to support conclusions? (NO)<br>-Are there concerns about ethical or regulatory requirements being met? (Yes. The authors need to include ethical statement in the manuscript)
**Results**<br><br>-Does the analysis presented match the analysis plan?<br>-Are the results clearly and completely presented?<br>-Are the figures (Tables, Images) of sufficient quality for clarity?	Does the analysis presented match the analysis plan?<br>-Are the results clearly and completely presented? (NO)<br>-Are the figures (Tables, Images) of sufficient quality for clarity? (NO)
**Conclusions**<br><br>-Are the conclusions supported by the data presented?<br>-Are the limitations of analysis clearly described?<br>-Do the authors discuss how these data can be helpful to advance our understanding of the topic under study?<br>-Is public health relevance addressed?	Are the conclusions supported by the data presented? (partly)<br>-Are the limitations of analysis clearly described? (No)<br>-Do the authors discuss how these data can be helpful to advance our understanding of the topic under study?<br>-Is public health relevance addressed? (Yes)
**Editorial and Data Presentation Modifications?**<br><br>Use this section for editorial suggestions as well as relatively minor modifications of existing data that would enhance clarity. If the only modifications needed are minor and/or editorial, you may wish to recommend "Minor Revision" or "Accept".	
**Summary and General Comments**<br><br>Use this section to provide overall comments, discuss strengths/weaknesses of the study, novelty, significance, general execution and scholarship. You may also include additional comments for the author, including concerns about dual publication, research ethics, or publication ethics. If requesting major revision, please articulate the new experiments that are needed.	General comments<br><br>The study by Adidja et al, addresses an important topic that could contribute to prevention and control of cholera globally during the ongoing COVID 19 pandemic. There are few studies which share the challenges, best Practices and Lessons learned from mass oral cholera vaccination campaign in urban Settings during COVID-19 pandemic. Adidja et al, conducted a cross sectional study that examined data collected between September 2020 and march 2021. While it is an interesting topic, there are major weaknesses with the manuscript as it stands as follows:<br><br>1. The authors state that the study design was a comparative cross-sectional study yet in the results and discussion sections little data and information is shared is to that effect.<br><br>2. The data presented and analysed were not performed to a high technical standard and are not described in sufficient detail. For example, most of the tables (tables 1-3) and figures (III.1-III.3) and lines 213-218 are percentages that

are not followed by numbers (numerator and denominator).

These two issues will need to be addressed to improve the readability and for the message by Adidja et al, to be understood by the readers.
Specific comments
1. Abstract. The abstract is not a true summary of the manuscript. For example, lines 27-30, " ……high case fatality rate of 4.3% ….", this information is not documented and referenced in the main text.

2. Abstract. The abstract is not structured according to the journal guidelines that require three subsections namely; Background, Methodology/Principal Findings, and Conclusions/Significance. The authors, will need to follow the journal abstract structure.

3. Authors summary. The authors did not include all components of the manuscript as required by the guidelines. The authors missed out the authors summary.

4. Introduction. Some statement such line 93-94, " the number of cholera cases considerately reduced after this first round" are not cited.

5. Introduction. Lines 95-95, " This second round took place in a particular context COVID-19 pandemic…. It would have more informative if the authors gave a brief information on status of COVID 19 in Cameroon or study area since the COVID19 appear in several sections of the manuscript.

6. Results. Lines 146-298, the numberings of the heading is a mixture of Roman and Arabic numerals and bullets which is difficult to comprehend by the readers. The authors need to select one the number format and use it throughout the manuscript.

7. Results. Lines 213-244, Adidja et al, include discussion in the results yet there is a separate section for discussion. The authors give citations in the results just like in the discussion. To improve the results, the authors will need to remove all statements that discuss results from the results section.

8. Results. Lines 164-167, the message in this figure is not clear to the readers. It is difficult to understand which component of the figure the authors wanted to magnify. The arrow for pullout is pointing to the boxed figure that has no legend. The panel with the legend, the legend is in French yet the manuscript is in English. The authors will need to clarify this information.

9. Results. Line 188, "figure III.4, Adverse events Following Immunization (AEFI)" it is not clear what the authors wanted to share in this diagram or table. There are abbreviations and codes which are not explained. The authors need to explain what is meant by D1-D6 and codes 0 and 1.

10. Results. Figure 2, "The graph below, which shows the number of people vaccinated by age group and sex, shows that the number of women vaccinated is much higher than the number of men". This graph is not the most appropriate to show the group with the highest number since the data are presented in opposite direction. Vertical bar graph would be more suitable to convey such information on comparison.

11. Discussion. Lines 312-319, 342-343, the authors keep on referring to COVID 19 context without giving enough background information on the extend of COVID 19 in the study area. since COVID 19 can spread from the health workers to the population and vise vasa. It would be more interesting if the authors gave a brief summary of COVID 19 in the study area or Cameroon. Refer to item no. 5 above.

12. Discussions. Lines 304-305, "We observed an 80.4% overall coverage for this campaign a marked increase from the 64% recorded during the first round in August 2020". The authors discuss results that were not presented in the data and they do not cite the source of the alien information. Adidja et al, will need to revise this section as well.

Confidential Comments to Editor

Comment here if you have any editorial concerns that should be relayed confidentially to the editor and/or journal staff. Comments entered in this field will NOT be communicated to the authors.

28th June 2021
Dear Editor (Dr. Mellisa Roskosky),

Re: Manuscript (PNTD-D-21-00790): Challenges, Best Practices and Lessons Learned from the first Mass vaccination campaign in Urban Settings of Cameroon in the COVID-19 era
Thank you so much for the wonderful opportunity to review this important topic on cholera prevention and control in Cameroon. I have found the task interesting and motivating to handle. I am available to provide any clarification. Thank you so much again. Sincerely, Godfrey Bwire

Competing Interests

Do you have any potential or perceived competing interests that may influence your review? Please review our Competing Interests policy and declare any potential interests that you feel the Editor should be aware of when considering your review.

No

Ethical Research and Reporting

Has all research been conducted to a high ethical standard, and is the work performed the same work which was approved? (Note that PLOS NTDs staff runs an internal ethics check before papers proceed to review, so this is a secondary check.)

All research involving human participants must have been approved by the authors' Institutional Review Board or an equivalent committee, and all clinical investigation must have been conducted according to the principles expressed in the Declaration of Helsinki. Informed consent, written or oral, should also have been obtained from the participants. A brief overview of the ethics reporting should be provided in the Methods; if no consent was given, the reason must be explained.

No

If you responded "No" to the previous question, please briefly outline your concerns here:

Not stated in the information shared. The authors will need to provide an ethical statement since data shared shared is on humans. This statement can be on IRB approval or exemption.

Data Availability

Have the authors made all data underlying the findings in their manuscript fully available?

The PLOS Data policy requires authors to make all data underlying the findings described in their manuscript fully available without restriction, except in cases where the data are legally or ethically restricted (for example, participant privacy is an appropriate restriction).

No

English Language

Is the manuscript presented in an intelligible fashion and written in standard English?

No

If you responded "No" to the previous question, would additional copyediting make this manuscript acceptable for publication?

No

Would this research be of interest to the general public? If so, we may consider arranging a press release for this article. Please provide reasons for your answer.

Yes: My comments are included in general summary.

PLOS authors have the option to publish the peer review history of their article (what does this mean?). If published, this will include your full peer review and any attached files.

If you choose "no", your identity will remain anonymous but your review may still be made public.

Do you want your identity to be public for this peer review? For information about this choice, including consent withdrawal, please see our Privacy Policy.

Yes: Godfrey Bwire

Do you want to get recognition for this review on Publons?

If you opt in, your Publons profile will automatically be updated to show a verified record of this review in full compliance with the journal's review policy. If you don't have a Publons profile, you will be prompted to create a free account.

Yes

Attachments:

Action	Description	File Name	Size	Last Modified
Download	Review report for the manuscript Number PNTD-D-21-00790	Review report_ Manuscript PNTD _D_21_00790_Godfrey Bwire.docx	19.9 KB	Jun 28 2021 5:33AM

Back   Edit Review   Print   Submit Review to Editorial Office
