# Supplementary material for: Case-area targeted preventive interventions to interrupt cholera transmission: Current implementation practices and lessons learned
Source: PLoS Negl Trop Dis. 2021 Dec 17;15(12):e0010042. doi: 10.1371/journal.pntd.0010042 (PMC8719662; doi:10.1371/journal.pntd.0010042)
Supplement: Attachment — Submitted filename: Reviewer report _ Manuscript PNTD-D-21-00779_Godfrey Bwire 25.10.pdf [file pntd.0010042.s004.pdf]

25th October 2021

Dear Editor PNTD (Attention: Dr. Jade Benjamin-Chung),

Re: Manuscript PNTD-D-21-00779, titled, "Case-area targeted preventive interventions to interrupt cholera transmission: current implementation practices and lessons learned."

Thank you once again for giving me the second opportunity to review the manuscript PNTD-D-21-00779, Case-area targeted preventive interventions to interrupt cholera transmission: current implementation practices and lessons learned. The manuscript has improved however, there are still important issues with the method section that can affect replicability by other researchers. For example, there is inconstancies in the timeframe. While the authors state that they focused in the past 10 year, the timeline is approximately 11 years and if the case studies are included it is 12 years. Furthermore, the authors also included two studied from 2004 (Cameroon, Reference 11) and 2008 (Kenya, Reference 12) It is important that these glaring issues are addressed. I have included this important information in the detailed comments attached. Therefore, though there is improvement on the manuscript and in the original version my recommendation was minor revision, given the choices I have to make in EM, I have kept this manuscript under the recommendation of the minor revision.

I am available to provide any other required information and clarification.

Thank you so much again.

Yours sincerely,,

Dr. Godfrey Bwire, MBChB, MPH, PhD

Reviewer's report

Reviewer: **Godfrey Bwire**

Manuscript No: **PNTD-D-21-00779**

Manuscript title: **Case-area targeted preventive interventions to interrupt cholera transmission: current implementation practices and lessons learned**

General comments

The authors have made efforts to address the issues raised with the original manuscript. This revised version is clearer and the study undoubtedly has the potential to streamline and strengthen CATI implementation due to the good recommendations listed by the authors. I applaud the authors for these well thought recommendations. However, in this updated version there are still important issues in the method section that need to be clarified to increase readability and allow for replicability of the study findings by other researchers.

Essential comments

1. **Method section.** Lines 105-107, "The search was limited to publications between January 2009 and November 2019; English language publications were included in both searches, in addition to French and Spanish publications in the grey literature search." and lines 96-97, " A mixed-methods approach to study CATI implementation was employed, including: 1) reviews of peer reviewed journal publications and grey literature published in the past ten years; .

   The authors state that the study was limited to ten years however this is approximately 11 years. Furthermore, lines 164, table 1, the authors included studies in Cameroon, Duala, 2004 Reference 11 and in Kenya, Nyanza, 2008,

Reference 12. This information that is not clear on the period of literature included makes it difficult for replicability of this study findings by other researchers. Therefore, the authors should revise the paper and clear inconstancies/ inaccuracies so as to make this study easily replicable by other researchers. The studies in Kenya and Cameron that are clearly out the period stated should be omitted.

2. **Abstract and method sections.** Lines 40-42, " **Conclusions/Significance**: CATIs are believed to be effective in reducing cholera outbreaks, but there is limited and context specific evidence of their effectiveness in reducing the incidence of cholera cases and lack of guidance for their consistent implementation.". in this statement, the author note that there is limited evidence on effectiveness of CATI yet there are other studies such that by Bompangue et al 2020, https://doi.org/10.1186/s12879-020-4916-0. are not included. Yet, lines 129-131, "Retrospective case studies were used to investigate CATI implementation in DRC (2017-2020), Haiti (2010-2019), Yemen (2016-2020), and Zimbabwe (2018-2019) where the approach was implemented to control cholera outbreaks. Locations were selected in …" included the period 2020.. Therefore to avoid misinterpretation of the selective use of literature and information, the authors should revise this manuscript and include this study or in their discussion should refer to it as new finding that have weakened/overshadowed their study findings.

3. **Abstract.** Lines 40-42, " **Conclusions/Significance**: CATIs are believed to be effective in reducing cholera outbreaks, but there is limited and context specific evidence of their effectiveness in reducing the incidence of cholera cases and lack of guidance for their consistent implementation.". my main concern with this conclusion is that the authors use the term "believed" where there is clear and robust study conducted by a competent team in a place (Bangladesh) that has shapped the current knowledge on the epidemiology of cholera (Sack et al, https://doi.org/10.1093/infdis/jiab440). Therefore, the authors should revise the statement and remove the word believe since the facts are available. This revision should be carried out in the entire manuscript where the term believed is used.

4. **Abstract and method sections. Abstract** Lines 29-32, " We identified 11 peer-reviewed and eight grey literature articles documenting CATIs and completed 30 key informant interviews in case studies in Democratic Republic of Congo, Haiti, Yemen, and Zimbabwe. We documented 15 outbreaks in countries where CATIs were used.". and me**thod section lines 128-135.** All 100% of the countries listed in this statement are fragile states. Further, majority of the studies shaping the finding were from the fragile states (https://thedocs.worldbank.org/en/doc/179011582771134576-0090022020/original/FCSFY20.pdf). Fragility is known to affect social services

even when effective approaches are applied (doi: 10.1093/heapol/czz142; https://www.usip.org/sites/default/files/resources/SR_301.pdf: http://www.gsdrc.org/docs/open/con86.pdf). When the study in Kenya (2008) is excluded, this effect becomes even more clearer. Therefore, could it be that the observed results are due to fragile nature of the states where studies were carried out? The authors will need to explain in the discussion section the effect of this on their findings.

Other comments

1. Lines 96-99,

   "A mixed-methods approach to study CATI implementation was employed, including: 1) reviews of peer reviewed journal publications and grey literature published in the past ten years; and 2) four retrospective case studies of recent cholera outbreaks in the Democratic Republic of the Congo (DRC), Haiti, Yemen, and Zimbabwe"

   and lines 29-32,

   " **Methodology/Principle Findings**: We investigated implementation of cholera case-area targetedinterventions (CATIs) using systematic reviews and case studies. We identified 11 peer-reviewed and eight grey literature articles documenting CATIs and completed 30 key informant interviews in case studies in Democratic Republic of Congo, Haiti, Yemen, and Zimbabwe. We documented 15 outbreaks in countries where CATIs were used"

   The authors use the term "recent" that is open to misinterpretation by readers and scientists interested in replicability of the study findings. Therefore, the authors should clearly specify the timeframe/period to allow for replicability of the findings.

2. In reference my earlier comment (first comment) on the original version which is not fully addressed yet. The authors interpreted rapid response team as CATI, yet this is WHO-AFRO strategy for outbreak investigation and response that is applicable to any infectious disease epidemic in WHO African region. Since most the studies are from WHO Afro region, I think that it would be important if the authors could raise this as a limitation in the interpretation of the study findings and conclusion.
