# Supplementary material for: Case-area targeted preventive interventions to interrupt cholera transmission: Current implementation practices and lessons learned
Source: PLoS Negl Trop Dis. 2021 Dec 17;15(12):e0010042. doi: 10.1371/journal.pntd.0010042 (PMC8719662; doi:10.1371/journal.pntd.0010042)
Supplement: Attachment — Submitted filename: Response to reviewers Oct 1 2021.docx [file pntd.0010042.s003.docx]

Journal: PLOS Neglected Tropical Diseases

Manuscript ID: PNTD-D-21-00779 - [EMID:596d04b94744c46d]

Title: "Case-area targeted preventive interventions to interrupt cholera transmission: current implementation practices and lessons learned"

Author(s): Mustafa Sikder, Chiara Altare, Shannon Doocy, Daniella Trowbridge, Gurpreet Kaur, Natasha Kaushal, Emily Lyles, Daniele Lantagne, Andrew S. Azman, Paul Spiegel

First, we would like to thank the reviewers for the comments and suggestions. The reviews have been very helpful in improving our manuscript, and we are happy to submit the revised version with response to reviewer comments herein. Please note the reviewers’ initial comments are in black, and our responses are indented and in blue; all line number reference are to the revised version of the manuscript.

# Editorial and Data Presentation Modifications

## Reviewer #1:

1. For the purpose of reviewing the contents of the manuscript, it will be more easier for the reviewers if the authors included line numbering on each of the sentences.

Thank you, we added line numbers.

## Reviewer #2:

## Abstract

2. I recommend against citing the first reference from Ali et al. even if this paper has been widely cited. Indeed, it provides doubtful estimates of the global burden of cholera based on unreliable extrapolations from only 3 limited cross-sectional cholera prevalence surveys!

Thank you for the suggestion. We understand the reviewers concern about the estimates. The Methods section of the article reports conducting a “…systematic search of all publicly available cholera case and fatality data…” and including multiple data sources such as WHO’s Weekly Epidemiology Records, PubMed, Global Infectious Disease and Epidemiology Network, and the Program for Monitoring Emerging Diseases.

Therefore, we consider referring to this article will strengthen our introductory statement that cholera is concentrated in low-resource, fragile, and humanitarian settings.

3. I suggest authors cite yearly WHO records even though they are likely underestimates.

Thank you. If this comment is related to the previous comment, both Ali et al. 2015 and Lessler et al. 2018 used WHO records to produce the estimates. Therefore, we think citing individual WHO records will be redundant in this case.

4. In their conclusion, authors write that CATI « are believed to be effective in reducing cholera outbreaks, but there is insufficient evidence in their effectiveness ». I do not agree with this statement as in « Evaluation measures varied, however, few evaluated cholera transmission reduction » : remove « however » ?

We added “…in reducing the incidence of cholera cases.” to clarify the limited evidence related to cholera incidence reduction and removed “however,”. (Lines 41-42)

## Introduction:

5. Authors should rather cite the number of cholera suspected cases recorded by WHO rather than this

As mentioned above, we believe the two reference papers that were produced using WHO’s reports should provide necessary reference to support our statement “Cholera remains a significant cause of mortality and morbidity, particularly in low-resource, fragile, and humanitarian settings.”.

## Methods

6. Rather than citing the Cochrane reference [5], authors could directly cite the PRISMA 2020 statement of the EQUATOR Network (<https://nam02.safelinks.protection.outlook.com/?url=https%3A%2F%2Fwww.equator-network.org%2Freporting-guidelines%2Fprisma%2F&amp;data=04%7C01%7Cpbspiegel%40jhu.edu%7Ccb7c153489d0449f903108d96ef1e2bc%7C9fa4f438b1e6473b803f86f8aedf0dec%7C0%7C0%7C637662808516691791%7CUnknown%7CTWFpbGZsb3d8eyJWIjoiMC4wLjAwMDAiLCJQIjoiV2luMzIiLCJBTiI6Ik1haWwiLCJXVCI6Mn0%3D%7C1000&amp;sdata=OmI1qXnXKH%2BLGMZ7v6mmgPRHKcVccvNGsssPSNvdg%2Bg%3D&amp;reserved=0>)

We updated the reference with the original PLOS Medicine article. (Reference # 6)

7. The PRISMA checklist is missing : <https://nam02.safelinks.protection.outlook.com/?url=https%3A%2F%2Fwww.equator-network.org%2Freporting-guidelines%2Fprisma%2F&amp;data=04%7C01%7Cpbspiegel%40jhu.edu%7Ccb7c153489d0449f903108d96ef1e2bc%7C9fa4f438b1e6473b803f86f8aedf0dec%7C0%7C0%7C637662808516691791%7CUnknown%7CTWFpbGZsb3d8eyJWIjoiMC4wLjAwMDAiLCJQIjoiV2luMzIiLCJBTiI6Ik1haWwiLCJXVCI6Mn0%3D%7C1000&amp;sdata=OmI1qXnXKH%2BLGMZ7v6mmgPRHKcVccvNGsssPSNvdg%2Bg%3D&amp;reserved=0>

Thank you, we provided the PRISMA flow diagram as a supplemental figure (Figure 1) which depicts the number of articles included/excluded at each step of the review process.

## Results

8. The process of outbreak selection is not clear to me. Did authors only select outbreaks they found CATI reports on? Please clarify

Thank you. As reported in the paper, we selected the sites consulting with the strategic advisory group. We further clarified that by including “…where the approach was implemented to control cholera outbreaks.”. (Lines 131-132)

9. Similarly, I do not understand whether the references cited in Table 1 only intend to describe the listed epidemics, or the CATI implemented strategies. In the latter case, and for example
a. for Haiti, Table 1 could directly include ref [21] and [27]

Table 1 includes the outbreak locations and reporting dates that were identified in the peer-reviewed (column 1) and grey literature (column 2) search and mentioned in the case study interviews (column 3). For Haiti, we included Ref 21 and 27 (revised numbers in the current version are 23 and 22) together with other references because all those articles were reviewed to prepare the manuscript.

b. For RDC, authors should include the report from Bompangue et al 2020 (<https://doi.org/10.1186/s12879-020-4916-0>).

Thank you. We did not include the paper because it was not published when we conducted the literature review, hence it did not meet our inclusion criteria. However, during our case studies we interviewed one of the main authors of the paper and included the lessons learned during the outbreak response.

c. For Sierra Leone and Guinea in 2012, authors should include this report : <https://nam02.safelinks.protection.outlook.com/?url=https%3A%2F%2Fplateformecholera.info%2Fattachments%2Farticle%2F394%2F5-GCSL_2012_SLL_ACF_001.pdf&amp;data=04%7C01%7Cpbspiegel%40jhu.edu%7Ccb7c153489d0449f903108d96ef1e2bc%7C9fa4f438b1e6473b803f86f8aedf0dec%7C0%7C0%7C637662808516691791%7CUnknown%7CTWFpbGZsb3d8eyJWIjoiMC4wLjAwMDAiLCJQIjoiV2luMzIiLCJBTiI6Ik1haWwiLCJXVCI6Mn0%3D%7C1000&amp;sdata=NmSWY4l5LE5CrHqWVDgIuXxPwRwKvrWX%2FP5TbU4Vj2k%3D&amp;reserved=0>

Thank you. That report did not come up in our search. We have read the report and it is interesting but doesn’t have an effect on the paper itself. Since it did not come up in our search, we have decided not to include it in the article.

d. A first description of cholera rapid response teams was published in 1971 by Voelkel (<https://nam02.safelinks.protection.outlook.com/?url=http%3A%2F%2Fwww.ncbi.nlm.nih.gov%2Fpubmed%2F5576843&amp;data=04%7C01%7Cpbspiegel%40jhu.edu%7Ccb7c153489d0449f903108d96ef1e2bc%7C9fa4f438b1e6473b803f86f8aedf0dec%7C0%7C0%7C637662808516701785%7CUnknown%7CTWFpbGZsb3d8eyJWIjoiMC4wLjAwMDAiLCJQIjoiV2luMzIiLCJBTiI6Ik1haWwiLCJXVCI6Mn0%3D%7C1000&amp;sdata=B9JWFi3ksXVKxl4p%2FMXqVxzIhRU3u7N2cwgq8mKR0f0%3D&amp;reserved=0>)

Thank you. We have found the paper, and since this is not related to our searches, dates and inclusion criteria, we have included it in our introduction. It is a very interesting paper indeed.(Ref # 5)

e. A CATI strategy was also implemented in 1998-1999 in Comoros with support of the Medecins du Monde NGO (<https://nam02.safelinks.protection.outlook.com/?url=https%3A%2F%2Fdevsante.org%2Farticles%2Fl-epidemie-de-cholera-aux-comores&amp;data=04%7C01%7Cpbspiegel%40jhu.edu%7Ccb7c153489d0449f903108d96ef1e2bc%7C9fa4f438b1e6473b803f86f8aedf0dec%7C0%7C0%7C637662808516701785%7CUnknown%7CTWFpbGZsb3d8eyJWIjoiMC4wLjAwMDAiLCJQIjoiV2luMzIiLCJBTiI6Ik1haWwiLCJXVCI6Mn0%3D%7C1000&amp;sdata=Zdu5rne8jxGJykZI9sZ8SxB5P1GWNwpLb8%2F%2F2YZ%2BgtA%3D&amp;reserved=0>)

Thank you, the article was published in 1998 and our review restricted the articles published between January 2009 and November 2019.

10. Page 9, ref [14] was published in 2017 and does not describe 2013-2019 CATI strategy in Haiti. Authors could rather cite ref [21] or [27] , or a more recent letter (<https://doi.org/10.1016/s2214-109x(20)30430-7>).

Ref 14 (revised Ref # 15) published in 2017 discussed the outbreak response in Juba, South Sudan not Haiti.

11. Please note that the study [20] describes a limited strategy implemented in Port-au-Prince in 2010-2011, which was not part of the nationwide CATI strategy launched in 2013. Further citations of this ref [20] are thus often ambiguous.

We included all studies that met the inclusion criteria for the systematic literature review. As the response implemented cholera case targeted interventions in an outbreak setting, we included it in the manuscript. This study was not cited as a reference to the 2013 nationwide CATI strategy.

12. page 10: please note that although the Haiti epidemic occurred 10 months after the earthquake, the disease was imported by a UN peace keeping battalion in a rural area (Mirebalais commune) and exploded 80km downstream of a highly likely contaminated river in a rice field plain, both of which had not been affected by the earthquake at all. Please modify the sentence as it wrongly suggests that cholera may have been triggered by the earthquake.

We included “…several months after the earthquakes.” to clarify. (Line 180)

13. Similarly, are authors sure that the 2016 cholera epidemic in Nepal was influenced by the April 2015 earthquake ?

We reported “…outbreaks in Haiti and Nepal occurred during earthquake recovery.”, which does not imply the earthquakes influenced the cholera outbreaks; it only reports the overlap of the earthquake recovery period with the cholera outbreak. Nevertheless, we understand the statement can confuse readers and thus clarified including “…several months after the earthquakes.” (Line 180)

14. page 13: there was no vaccinator in the CATI teams in Haiti as no OCV was administered (Table 2). Please add « respectively »

We did not report OCV for Haiti in Table 2.

15. page 13: please explain what were the mixed teams in Haiti

Thank you, we added “… mixed teams, comprised of health and WASH staff”. (Line 221)

16. page 14 : as described in ref [27], a specific weekly alert system at the communal level was initiated in Haiti in 2013 in order to help prioritize and monitor CATIs. This system preceded the generalization of case line list sharing. Authors should add this information.

Thank you, we added “CATIs and community level alert systems were used to prioritize response.” (Line 234-235)

17. page 14: in Haiti, a nation-wide use of RDT was implemented from 2013 in order to help prioritizing CATIs. This strategy was later abandoned as RDT results were not readily available for RRTs and their results proved unreliable.

Thank you for the information. The articles we reviewed did not report about the unreliable RDT results and the change in testing policy, hence we are not able to include this information. If the reviewer could provide a reference from a peer-reviewed article, we will be happy to include this information.

18. page 14: results from stool culture were also used for CATI implementation in Haiti during the last years of the strategy: as part of the elimination process, RRTs were asked to implement a second CATI in case of positive culture

Same as above, these are important lessons learned and we will include those if the reviewer could direct us to the appropriate references from peer-reviewed articles.

19. page 15: please add references to « 4) timely access to case information to rapidly implement CATI ». For instance ref [27] was cited for the same topic on page 14.

Thank you, we added the reference. (Revised ref # 10, 22)

20. page 17: in Haiti, coverage was also reported as the proportion of responded cholera alerts (see ref [27] : 49% between July 2013 and June 2017).

Thank you, we added “…in terms of responded cholera alerts (49%),[28]”. (Line 297-298)

21. page 17: the UNICEF’s global review of cholera rapid response teams reports a monthly cost of $10,234 per team in Haiti (not $180!), and $2,400-3,000 in Yemen (not $1,776). Please check and correct.

Thank you, we corrected the figure for Haiti, however, we checked the reported cost for Yemen was correctly reported in our paper. (Line 305)

22. in « differences in cholera incidence between those that received a timely CATI », the term « those » is imprecise.

Thank you, we replaced “those” with “the households”. (Line 310)

23. page 16-18: I recommend to present behavior change results (that can be considered as CATI outcomes), before effectiveness and impact results. The global « theory of change » scheme could be used to better arrange this part of the review (see for instance <https://nam02.safelinks.protection.outlook.com/?url=http%3A%2F%2Ffic.tufts.edu%2Fassets%2FWASH-Systematic-Review.pdf&amp;data=04%7C01%7Cpbspiegel%40jhu.edu%7Ccb7c153489d0449f903108d96ef1e2bc%7C9fa4f438b1e6473b803f86f8aedf0dec%7C0%7C0%7C637662808516701785%7CUnknown%7CTWFpbGZsb3d8eyJWIjoiMC4wLjAwMDAiLCJQIjoiV2luMzIiLCJBTiI6Ik1haWwiLCJXVCI6Mn0%3D%7C1000&amp;sdata=n%2FoCUFjJD9G6kneTdmt6F3QBLERAEqa7S5lNbIzzJdU%3D&amp;reserved=0>)

Thank you for the suggestion. We presented behavior change results after timeliness, coverage, and cost to follow the order of CATI implementation process.

We are concerned that bringing the behavior change results at the beginning – which are produced once CATIs are implemented – will confuse the readers.

Additionally, we would like to add that the behavior change results are reported before the outputs and impacts of the interventions.

## Discussion

24. page 19 : I do not understand the meaning of « which reported formative research to design the intentions ». I do not get the specificities of this very good study [23] on that aspect.

The interventions for the study were the selected from previous formative result. We clarified by adding “…which reported three months of piloting and previous formative research to design the intentions,”. (Line 353-354)

25. page 22: considering the proposed definition of CATI, do authors consider that interventions targeting a cholera cluster (e.g. a neighborhood, a locality or even a commune) could be considered as a CATI ?

As proposed in the article, both household and community level interventions can be considered as CATIs because the required intervention coverage to effectively interrupt the transmission can substantially vary depending on several factors related to the outbreak location.

26. no paragraph on the limitations of the study

The penultimate paragraph in the Discussions lists the limitation of our research. Paragraph starts on (Line 411).

## References :

27. ref [8]: URL does not seem to work

Added the URL

28. ref [18]: URL does not work

Added the URL

29. ref [19]: URL does not work

Added the URL

30. ref [20]: URL does not work

We provided a DOI in the reference (doi:10.2166/wh.2013.050).

31. ref [25]: URL does not work

Added the URL.

32. ref [28]: URL does not work

Added the URL

33. ref [31]: URL does not work

We provided a DOI (doi:10.1371/journal.pone.0226549)

# Summary and General Comments

## Reviewer #1:

This is well written manuscript on a valuable study that synthesizes available literature on CATI as a strategy for prevention and control of cholera outbreaks. Most importantly, the authors investigated and described weaknesses with previous implementation of CATI and give evidence based recommendation to strengthen CATI intervention implementation. The study has the potential to increase scientific knowledge on CATI.

However, there are still few important weaknesses in the content that need to addressed and are essential to improve clarity of the message, remove ambiguity in the understanding by the readers and enhance the quality of the contents therein.

We sincerely thank the reviewer for carefully reviewing the draft and for providing useful suggestions.

34. The major flaws with this manuscript are in the categorization and inclusion of the studies using the term RRT as synonymous to CATI interventions. Yet CATI and RRT are two distinct terms that are used differently by the cholera outbreak responders/ stakeholders. Also, given that seven out of eleven countries in this paper (table 2.) were from sub-Saharan Africa where cholera prevention is guided by the implementation of Integrated Disease Surveillance and Response (World Health Organization. Technical Guidelines for Integrated Disease Surveillance and Response in the WHO African Region THIRD EDITION. 2019. Available: https://nam02.safelinks.protection.outlook.com/?url=https%3A%2F%2Fapps.who.int%2Firis%2Fbitstream%2Fhandle%2F10665%2F325015%2FWHO-AF-WHE-CPI-05.2019-eng.pdf&amp;data=04%7C01%7Cpbspiegel%40jhu.edu%7Ccb7c153489d0449f903108d96ef1e2bc%7C9fa4f438b1e6473b803f86f8aedf0dec%7C0%7C0%7C637662808516701785%7CUnknown%7CTWFpbGZsb3d8eyJWIjoiMC4wLjAwMDAiLCJQIjoiV2luMzIiLCJBTiI6Ik1haWwiLCJXVCI6Mn0%3D%7C1000&amp;sdata=3WDNiWPB7C47%2F1MOsjsAj8tidUGe9GdVq9MALeCOU9E%3D&amp;reserved=0) use of RRT as synonymous to CATI has to be very cautious.

According to IDSR/WHO, A Rapid Response Team (RRT) is composed of experts who take the lead in conducting the initial investigation of reported and suspected cases or outbreaks so as to confirm the nature of the event under investigation. It is also the responsibility of the RRT to initiate the preliminary control/containment measures needed to prevent further spread of the disease. The same information is also articulated in the cholera prevention WHO RRT guide (World Health Organization. Guide for Rapid Response Teams (RRTs) for Cholera Outbreak investigation & initial response. 2010. Available: https://nam02.safelinks.protection.outlook.com/?url=https%3A%2F%2Fplateformecholera.info%2Fattachments%2Farticle%2F672%2FRRT%2520%2520cholera%2520outbreak%2520investigation%2520guide.pdf&amp;data=04%7C01%7Cpbspiegel%40jhu.edu%7Ccb7c153489d0449f903108d96ef1e2bc%7C9fa4f438b1e6473b803f86f8aedf0dec%7C0%7C0%7C637662808516701785%7CUnknown%7CTWFpbGZsb3d8eyJWIjoiMC4wLjAwMDAiLCJQIjoiV2luMzIiLCJBTiI6Ik1haWwiLCJXVCI6Mn0%3D%7C1000&amp;sdata=1W8PejORF6DI%2BgUwoWo77HhAJtSy9%2FrTqzIH1q7fLvc%3D&amp;reserved=0).

In summary RRT is constituted by experts and there role is to do the initial investigation and response and can be in place before, during and after the cholera outbreak.

While CATI is one of the cholera control strategy that can be used by the RRT to contain/ interrupt the outbreak. Therefore, Mustafa et al, should revise manuscript and exclude studies on RRT.

Thank you for the comment. We agree with the reviewer that CATI and RRT are two different mechanism and should not be used synonymously. However, we found, particularly in the grey literature, that CATI and RRT were interchangeably used to describe case area targeted cholera control interventions, and thus we were flexible in reviewing reports which may not have explicitly used the term “CATI” but implemented a cholera case/cluster of cases targeted interventions. We reviewed every instance of RRT and excluded those that were just an expert team conducting initial assessment in emergencies and included when that was cholera CATI.

The sole purpose was to widen the inclusion criteria to capture lessons learned which otherwise would have been excluded if we only restricted to “CATI” as the inclusion criteria. Furthermore, to standardize the terminology, at the Discussions we proposed a definition to describe CATIs in future reporting.

35. Introduction. Page 4, second paragraph, “As the terms CATI and Rapid Response Teams (RRT) were interchangeably used, and at the time CATI approaches were used, but the term itself was not used, we used a broad definition of CATI to examine the evidence: cholera case targeted interventions to interrupt transmission of the disease at the household and/or community level” according to IDSR/WHO the term CATI and RRT mean different things. This has the potential to confuse the reader who have accessed IDSR/WHO guidelines. The authors will need to revise this section and possibly exclude studies with RRT from the manuscript.

Thank you. As mentioned above, the search term deliberately included both CATI and RRT to ensure we don’t exclude any document which implemented a CATI approach but did not use the specific term. However, the review did not include any reports that were not cholera case targeted interventions. We clarified this by adding “RRT articles that did not employ cholera case targeted response teams or cholera case targeted interventions were not included in this review.”. (Lines 91-92)

36. Method section. Page 6, second paragraph, “ Both the included peer and grey literature were categorized as reporting on CATI or other rapid response interventions). This statement is related to above on RRT. Furthermore, other rapid response interventions is open to speculation and misinterpretation. Hence, Mustafa et al, will need to revise this section to remove possible confusion of the readers.

Thank you, we did not include other RRT reports that were identified as part of the larger review process. We clarified this by adding “…and only CATI interventions were included in this review.” (Line 123)

37. Result section. Page10, Table 2. The authors use the terms health and surveillance to categorized activities of CATI. However, the activities listed in surveillance can still fit in health. The authors could therefore merge the two under one sub-heading.

We understand some health and surveillance activities in cholera outbreak responses can overlap. However, we do not consider that merging the two categories will be useful to improve the clarity. Instead, it may confuse the readers since most of those activities are pretty clearly health or surveillance activities.

38. Discussion. Page 18, first paragraph, “ We noted many ambiguities and inconsistencies regarding RRTs and CATIs in terms of definitions, interventions combinations and implementation strategies, integration, coordination, data sharing, and reporting across outbreaks”. The author appreciates that there were many ambiguities for RRTs and CATIs. However, in the subsequent discussion the authors only focus on CATIs and nothing is given on RRTs. This is yet another important reason for Mustafa et al, to revise the contents of the paper in the introduction and methodology section and focus on CATIs as reflected in the title, abstract, discussion and conclusion of this manuscript.

Thank you. In the subsequent sections of the Discussions, we focused on CATIs because we only studied CATIs in this paper. We addressed the concerns the reviewer mentioned about the CATI and RRT earlier (comments 34-36) and hope the paper is clear in this version.

39. Discussion. Page 20, first paragraph, “The scientific evidence of the effectiveness of CATIs in reducing cholera transmission is limited. A recent scoping review found some evidence that antibiotic chemoprophylaxis, single-dose OCV, intensive hygiene promotion, and point-of-use water treatment to rapidly limit cholera transmission in the household and its high-risk radius”. It is clear what message the authors wanted to share. The first sentence points to lack or limited evidence while the second sentence show that there is evidence for effectiveness. Hence, the authors will need to revise this paragraph.

Thank you. We wanted to clearly state that the evidence is limited, but not absent. Therefore, we first said the evidence is limited and then reported a summary of that limited evidence which were only few interventions of a typical CATI.

To clarify further, we added “A recent scoping review reported evidence of some CATI activities such as, antibiotic chemoprophylaxis…”. (Line 360-361)

Other comments

40. Page 15, paragraph 2, “ Haiti,[20] and Bangladesh[23] the interventions started at case admission or discharge where, for example, antibody prophylaxis and hygiene..” The authors should review the highlighted word and if necessary replace it.

Thank you. We revised the sentence as: “In Cameroon,[11] DRC,[32] Haiti,[23] and Bangladesh[25] the interventions started at case admission or discharge where antibody prophylaxis and hygiene kits were distributed to the case’s household”. Line 287-288

41. Page 16, some abbreviations such as CTC are not explained. The authors should review the manuscript and ensure that all abbreviations are explained to avoid misinterpretation by the readers.

Thank you, we added the full form of the acronym for CTC (Line 192) and NGO (Line 157) in their first appearance.

Note that READY is not an acronym, but rather it refers to Global readiness for major disease outbreak response.

## Reviewer #2:

In this very interesting mixed-methods study using a review of published peer-reviewed and grey literature, authors address the implementation of case-area targeted interventions against cholera.

This piece of work is important as such cholera control strategy has been implemented in several major cholera epidemic over the past decade.

The manuscript is well written, the amount of aggregated information is important, authors propose highly relevant comments and several valuable recommendations, and they should be acknowledged for this piece of work.

Nevertheless, the manuscript suffers from the absence of several important publications and reports, and from several imprecisions and discrepancies which are listed in my specific comments and should be corrected.

Thank you. We sincerely thank the reviewer for recognizing the importance of the work and providing useful suggestions to improve the manuscript. We have addressed the concerns raised by the reviewer in the revised version.

Besides, I do not support one aspect of the overall conclusion of this review, which on several occasions implies that CATI effectiveness has not yet been established and that promotion of CATI would so far only be a matter of expert opinion. Authors do cite a randomized control trial of hospital-based hygiene and water treatment intervention in Bangladesh [23] which showed that distributing cholera kits in hospital to the family of cholera cases reduced secondary cases. They also cite a quasi-experimental study conducted in Haiti [21], which found that prompt and repeated CATIs were associated with a significant reduction of cholera outbreak duration and cumulative case incidence. Besides, as authors wrote in a recent report of the John Hopkins University about the same subject as the present article

(https://nam02.safelinks.protection.outlook.com/?url=http%3A%2F%2Fhopkinshumanitarianhealth.org%2Fassets%2Fdocuments%2FRRT_CaseStudy_Report_2021.pdf&amp;data=04%7C01%7Cpbspiegel%40jhu.edu%7Ccb7c153489d0449f903108d96ef1e2bc%7C9fa4f438b1e6473b803f86f8aedf0dec%7C0%7C0%7C637662808516701785%7CUnknown%7CTWFpbGZsb3d8eyJWIjoiMC4wLjAwMDAiLCJQIjoiV2luMzIiLCJBTiI6Ik1haWwiLCJXVCI6Mn0%3D%7C1000&amp;sdata=LemJnCC3W4P8pX15JWH5PFBGuLCid6g00yXItL653m0%3D&amp;reserved=0), no cholera case has been confirmed in the Haiti since February 2019, which could represent a relevant proof of concept of the CATI approach, as this was implemented as a massive national strategy between 2013 and 2019, with no alternative explanation of cholera disappearance so far. Therefore, they should explain why they do not seem to consider this as as evidence of CATI effectiveness.

Thank you for the comment. We first want to clarify that we do not want to promote that CATI implementation should be “a matter of expert opinion”. We could not find the sentence(s) in the conclusion that alluded such interpretation and will be happy to revise that if the reviewer could direct us.

We wanted to emphasize that the effectiveness is limited and context specific. As the reviewer mentioned, the Bangladesh study was in a non-outbreak urban settings and administered from a hospital, none of which are conventional CATI settings. Additionally, the study population had better access to health care compared to a typical CATI site.

To our knowledge, Haiti is the only example where both health and WASH components of CATI were implemented at scale and reliable evidence in controlling cholera transmission was documented. Therefore, we wanted to focus on the limited evidence and revised by adding the term “context specific” to further clarify. (Lines 41 and 359-360)

Following a modern science-based approach (https://nam02.safelinks.protection.outlook.com/?url=https%3A%2F%2Fsciencebasedmedicine.org%2Fabout-science-based-medicine%2F&amp;data=04%7C01%7Cpbspiegel%40jhu.edu%7Ccb7c153489d0449f903108d96ef1e2bc%7C9fa4f438b1e6473b803f86f8aedf0dec%7C0%7C0%7C637662808516701785%7CUnknown%7CTWFpbGZsb3d8eyJWIjoiMC4wLjAwMDAiLCJQIjoiV2luMzIiLCJBTiI6Ik1haWwiLCJXVCI6Mn0%3D%7C1000&amp;sdata=%2BjdepPC1b1A5ekocYCtH85GWXWXseGboutVSLNd3BFQ%3D&amp;reserved=0), I thus invite authors to consider a less stringent definition of evidence production in such epidemic contexts than the sole randomized control trials. Water disinfection, hand washing, sanitation and so on have for a long time now been shown to prevent fecal-oral diseases such as cholera.

I thus strongly believe that the state of « clinical equipoise » is not present regarding the question of conducting a CATI or not, and all cases should now be targeted. As a matter of fact, many experts, field public health actors as well as political or administrative leaders do consider that randomizing which cholera case family should or should not receive a CATI during cholera epidemics raises serious ethical issues.

Thank you. We completely agree with the reviewer that a randomized control trial or similar experimental study to assess CATI effectiveness would be not only difficult but could also rise ethical concerns. We discussed this in the fourth paragraph of the Discussion. Considering this study design limitation, we want to add that there are operational research methodologies that can improve the current quality and generalizability of CATI effectiveness evidence. We added “Additionally, partnership between research institutions and emergency responders to systematically study the different components of the CATI implementation can also improve the reliability and generalizability of the evidence.” to clarify. (Lines 378-380)

Nevertheless, I strongly agree with authors that « The scientific evidence of the effectiveness of CATIs in reducing cholera transmission is limited » (page 20). I can only join them on the fact that « measuring and attributing effectiveness to one approach or one intervention during an outbreak » is highly complex. And I totally agree that « monitoring systems of responding organizations » should be strengthened: this is precisely what was performed in Haiti. Some additional evidence to optimize the composition of the CATI package, or the selection of neighboring households to be targeted is urgently needed, and I imagine that valuable randomized studies could be conducted to answer these questions.

Thank you. We agree that the CATI experience in Haiti is a useful source of many lessons learned. With this manuscript, we aim to spread those lessons learned.

I thus think that authors should modify several sentences using a less ambiguous wording such as:

42. conclusion of the abstract: « CATIs are believed to be effective in reducing cholera outbreaks, but there is insufficient evidence in their effectiveness ».

Thank you, we replaced “insufficient” with “limited and context specific” (Lines 41 and 359-360)

43. introduction: « CATI is conceptualized as an efficient way to interrupt cholera outbreaks…

We replaced that with “While CATIs are used to interrupt cholera outbreaks, …”. (Line 79-80)

44. Evaluation measures in results: « None of the documents reviewed evaluated the effect of individual interventions in reducing the number of new cases or interrupting the cholera transmission pathways ». This is wrong, as ref [23] demonstrated that distributing cholera kits in hospital to the family of cholera cases reduced secondary cases in Bangladesh, and ref [21] demonstrated that prompt and repeated CATIs were associated with a significant reduction of cholera outbreak duration and cumulative case incidence.

Thank you, both the Bangladesh and Haiti study reported the cholera incidence reduction because of the complete CATI intervention, but none associated individual interventions with the reduction. To clarify, we added “While the Bangladesh and Haiti studies reported about the complete CATI approach, none of the documents reviewed evaluated the effect of individual interventions (e.g. only chlorine tablet distribution or only awareness session etc.) in reducing the number…” (Line 278-280)

45. Similarly within the same sentence « there were no prospective evaluations of CATI’s effectiveness » : ref [23] was a prospective randomized control trial. And ref [21] was a quasi-experimental evaluation of CATI’s effectiveness which could also be considered as « prospective » : although analyses were retrospective and CATIs were not randomized, both the exposition (CATIs) and outcome (cholera suspected cases) were routinely and prospectively recorded

We removed this part of the sentence. (Lines 281-282)

46. Discussion: « … selection of interventions did not appear to be based on evidence of their effectiveness. »

In our review, except the Bangladesh study, we did not find the rationale for selecting the CATI interventions. We revised the sentence as “…, selection of interventions was not reported.” (Line 354-355)
