# Supplementary material for: Case-area targeted preventive interventions to interrupt cholera transmission: Current implementation practices and lessons learned
Source: PLoS Negl Trop Dis. 2021 Dec 17;15(12):e0010042. doi: 10.1371/journal.pntd.0010042 (PMC8719662; doi:10.1371/journal.pntd.0010042)
Supplement: Attachment — Submitted filename: Response to reviewers Nov 21 2021.docx [file pntd.0010042.s005.docx]

Journal: PLOS Neglected Tropical Diseases

Manuscript ID: (PNTD-D-21-00779R1) - [EMID:73b0fcf6095d558a]

Title: "Case-area targeted preventive interventions to interrupt cholera transmission: current implementation practices and lessons learned"

Author(s): Mustafa Sikder, Chiara Altare, Shannon Doocy, Daniella Trowbridge, Gurpreet Kaur, Natasha Kaushal, Emily Lyles, Daniele Lantagne, Andrew S. Azman, Paul Spiegel

First, we would like to thank the reviewers for the comments and suggestions. The reviews have been very helpful in improving our manuscript, and we are happy to submit the revised version with response to reviewer comments herein. Please note the reviewers’ initial comments are in black, and our responses are indented and in blue; all line number reference are to the revised version of the manuscript.

## Reviewer #1:

1. The authors have made efforts to address the issues raised with the original manuscript. This revised version is clearer and the study undoubtedly has the potential to streamline and strengthen CATI implementation due to the good recommendations listed by the authors. I applaud the authors for these well thought recommendations. However, in this updated version there are still important issues in the method section that need to be clarified to increase readability and allow for replicability of the study findings by other researchers.

Thank you for the comments. We addressed the concerns raised by the reviewer.

2. Method section. Lines 105-107, “The search was limited to publications between January 2009 and November 2019; English language publications were included in both searches, in addition to French and Spanish publications in the grey literature search.” and lines 96-97, “ A mixed-methods approach to study CATI implementation was employed, including: 1) reviews of peer reviewed journal publications and grey literature published in the past ten years; .

The authors state that the study was limited to ten years however this is approximately 11 years. Furthermore, lines 164, table 1, the authors included studies in Cameroon, Duala, 2004 Reference 11 and in Kenya, Nyanza, 2008, Reference 12. This information that is not clear on the period of literature included makes it difficult for replicability of this study findings by other researchers. Therefore, the authors should revise the paper and clear inconstancies/ inaccuracies so as to make this study easily replicable by other researchers. The studies in Kenya and Cameron that are clearly out the period stated should be omitted.

Thank you. We revised lines 99 as “reviews of peer-reviewed journal publications and grey literature published between January 2009 and November 2019;”.

Reference 11 was published in 2006. We included this study following the forward citation tracking method (as mentioned in lines 114). To further clarify that this study was not included directly from the search result but through forward citation tracking, we included the breakdown in lines 155-156: “(10 from direct search result and one from forward citation tracking)”. Reference 12 was published in 2013 and thus was identified by the literature search.

3. Lines 40-42, “Conclusions/Significance: CATIs are believed to be effective in reducing cholera outbreaks, but there is limited and context specific evidence of their effectiveness in reducing the incidence of cholera cases and lack of guidance for their consistent implementation.” In this statement, the author note that there is limited evidence on effectiveness of CATI yet there are other studies such that by Bompangue et al 2020, https://doi.org/10.1186/s12879-020-4916-0. are not included. Yet, lines 129-131, “Retrospective case studies were used to investigate CATI implementation in DRC (2017-2020), Haiti (2010-2019), Yemen (2016-2020), and Zimbabwe (2018-2019) where the approach was implemented to control cholera outbreaks. Locations were selected in …” included the period 2020.. Therefore, to avoid misinterpretation of the selective use of literature and information, the authors should revise this manuscript and include this study or in their discussion should refer to it as new finding that have weakened/overshadowed their study findings .

We respectfully disagree with the reviewer's characterization of our study as “…selective use of literature and information…”. The requested Bompangue et al 2020 article was not included in our literature search because it was published in March 2020 and our search end date was November 2019. Had published within our search timeframe, we would have gladly included the study since there are only a few peer-reviewed articles on case-targeted interventions. In this revised version, we referred to the Bompangue et al article in our Discussion lines 398-400: “Additionally, a study in Kinshasa, DRC reported the use of a targeted grid approach to delivering WASH interventions to contain the outbreak in urban settings.[37]”

4. Abstract. Lines 40-42, “ Conclusions/Significance: CATIs are believed to be effective in reducing cholera outbreaks, but there is limited and context specific evidence of their effectiveness in reducing the incidence of cholera cases and lack of guidance for their consistent implementation.”. my main concern with this conclusion is that the authors use the term “believed” where there is clear and robust study conducted by a competent team in a place (Bangladesh ) that has shapped the current knowledge on the epidemiology of cholera (Sack et al, https://doi.org/10.1093/infdis/jiab440). Therefore, the authors should revise the statement and remove the word believe since the facts are available. This revision should be carried out in the entire manuscript where the term believed is used.

We revised the sentence in line 40 as: “CATIs appear effective in reducing cholera outbreaks…”. Also removed “believe” from line 51.

5. Abstract and method sections. Abstract Lines 29-32, “ We identified 11 peer-reviewed and eight grey literature articles documenting CATIs and completed 30 key informant interviews in case studies in Democratic Republic of Congo, Haiti, Yemen, and Zimbabwe. We documented 15 outbreaks in countries where CATIs were used.”. and method section lines 128-135. All 100% of the countries listed in this statement are fragile states. Further, majority of the studies shaping the finding were from the fragile states (https://nam02.safelinks.protection.outlook.com/?url=https%3A%2F%2Fthedocs.worldbank.org%2Fen%2Fdoc%2F179011582771134576-0090022020%2Foriginal%2FFCSFY20.pdf&amp;data=04%7C01%7Cpbspiegel%40jhu.edu%7Cf6902f7fc6b943fb00a908d9a2be0e1e%7C9fa4f438b1e6473b803f86f8aedf0dec%7C0%7C0%7C637719760656710690%7CUnknown%7CTWFpbGZsb3d8eyJWIjoiMC4wLjAwMDAiLCJQIjoiV2luMzIiLCJBTiI6Ik1haWwiLCJXVCI6Mn0%3D%7C3000&amp;sdata=T4Um%2B6XM2XUWIleeALmmn1%2BL5I8D9A7szmU1DjdVw7o%3D&amp;reserved=0 ). Fragility is known to affect social services even when effective approaches are applied ( doi: 10.1093/heapol/czz142; https://nam02.safelinks.protection.outlook.com/?url=https%3A%2F%2Fwww.usip.org%2Fsites%2Fdefault%2Ffiles%2Fresources%2FSR_301.pdf&amp;data=04%7C01%7Cpbspiegel%40jhu.edu%7Cf6902f7fc6b943fb00a908d9a2be0e1e%7C9fa4f438b1e6473b803f86f8aedf0dec%7C0%7C0%7C637719760656710690%7CUnknown%7CTWFpbGZsb3d8eyJWIjoiMC4wLjAwMDAiLCJQIjoiV2luMzIiLCJBTiI6Ik1haWwiLCJXVCI6Mn0%3D%7C3000&amp;sdata=xBBh5lF7d32HyMqJuuyc6Lq0haP5AJZFcNPAT2umx84%3D&amp;reserved=0: https://nam02.safelinks.protection.outlook.com/?url=http%3A%2F%2Fwww.gsdrc.org%2Fdocs%2Fopen%2Fcon86.pdf&amp;data=04%7C01%7Cpbspiegel%40jhu.edu%7Cf6902f7fc6b943fb00a908d9a2be0e1e%7C9fa4f438b1e6473b803f86f8aedf0dec%7C0%7C0%7C637719760656710690%7CUnknown%7CTWFpbGZsb3d8eyJWIjoiMC4wLjAwMDAiLCJQIjoiV2luMzIiLCJBTiI6Ik1haWwiLCJXVCI6Mn0%3D%7C3000&amp;sdata=ocw898v5YTlBrjArbasnuozG%2FdNnommUivZOOftQ3EI%3D&amp;reserved=0 ). When the study in Kenya (2008) is excluded, this effect becomes even more clearer. Therefore, could it be that the observed results are due to fragile nature of the states where studies were carried out? The authors will need to explain in the discussion section the effect of this on their findings.

We agree that the fragile states are vulnerable to a range of internal and external shocks and that could have implications on the effectiveness of the CATI approach. However, such investigation is beyond the scope of the study. We included this limitation in lines 436-439: “Majority of the countries in the study were deemed to be fragile by the World Bank. [38] While studying the implications of countries fragility on the effectiveness of CATIs were beyond the scope of the manuscript, readers should consider this caveat when interpreting the results.”

6. Lines 96-99, “A mixed-methods approach to study CATI implementation was employed, including: 1) reviews of peer reviewed journal publications and grey literature published in the past ten years; and 2) four retrospective case studies of recent cholera outbreaks in the Democratic Republic of the Congo (DRC), Haiti, Yemen, and Zimbabwe” and lines 29-32, “Methodology/Principle Findings: We investigated implementation of cholera case-area targeted interventions (CATIs) using systematic reviews and case studies. We identified 11 peer-reviewed and eight grey literature articles documenting CATIs and completed 30 key informant interviews in case studies in Democratic Republic of Congo, Haiti, Yemen, and Zimbabwe. We documented 15 outbreaks in countries where CATIs were used”

The authors use the term “recent” that is open to misinterpretation by readers and scientists interested in replicability of the study findings. Therefore, the authors should clearly specify the timeframe/period to allow for replicability of the findings

Thank you. We revised lines 99-101 as: “1) reviews of peer-reviewed journal publications and grey literature published between January 2009 and November 2019; and 2) four retrospective case studies of cholera outbreaks in the Democratic Republic of the Congo (DRC) (2017-2020), Haiti (2010-2019), Yemen (2016-2020), and Zimbabwe(2018-2019).” Given the 300 words limit for Abstract of the journal, it will be difficult to clearly report all the timeframes/periods in the abstract and also keep the important findings of the study.

7. In reference my earlier comment (first comment) on the original version which is not fully addressed yet. The authors interpreted rapid response team as CATI, yet this is WHO-AFRO strategy for outbreak investigation and response that is applicable to any infectious disease epidemic in WHO African region. Since most the studies are from WHO Afro region, I think that it would be important if the authors could raise this as a limitation in the interpretation of the study findings and conclusion.

Thank you. We again wish to clarify that we did not interpret RRT as CATI. We reported in lines 86-95 – why and how we used a flexible definition to find studies that implemented a case area targeted intervention. We understand WHO-AFRO’s strategy. But these recommendations are **not** strictly followed when reporting cholera outbreak responses. Therefore, we had to use flexible initial search terms to identify all relevant reports. Then we used clear criteria to only include reports that reported a **case area targeted cholera control intervention**. We want to emphasize that we are eager to admit any limitations of our study, but we believe using a flexible definition to identify relevant reports for the initial screening was not a limitation of the study.

## Reviewer# 2

1. I sincerely acknowledge authors for their modifications and clarifications, and I believe I am now in line with the authors’ point of view.

Thank you for the comment.

2. In the conclusion of their abstract, I however think the verb « believe » wrongly suggests that CATI effectiveness is a matter of unscientific belief. I suggest a more neutral sentence, for instance like this : « CATIs appear effective in reducing cholera outbreaks, but the available evidence remains limited and context specific, and no guidance for their consistent implementation has been released so far . »

Thank you, we revised the line 40 as suggested: “CATIs appear effective in reducing cholera outbreaks…”

3. I understand the fact that the grey publication search was limited to the period January 2009 - November 2019. Considering the limited number of scientific publication on the subject, I really think that this period should be extended for them. In particular, the paper from Bompangue et al 2020 should really be cited in a systematic review published in 2022 and with a strong expected impact .

Thank you. We referred to Bompangue et al 2020 in Lines 398-400 as: “Additionally, a study in Kinshasa, DRC reported the use of a targeted grid approach delivering WASH interventions to contain the outbreak in urban settings.[37]”

4. I don’t understand the position of the new ref [5] (Voelkel 1971). Although it rapidly lists cholera transmission pathways, this French pioneering paper mainly describes principles of CATIs .

Thank you. A reviewer pointed us to this important article, and we think it is a valuable early account of CATI principles that strengthen our arguments.

5. I still strongly think that authors should also include the Sierra Leone and Guinea in 2012 report in their systematic review, even though it does not significantly change authors conclusions. This report provides valuable data on CATI implementation and impact, and to be systematic, this review should definitely cite this report .

Thank you, we added this report. Lines 159-162: “Additionally, one document was included during the peer-review process following one of the reviewer’s suggestion.”. We also updated the subsequest reviewed documents numbers and country lists in the remaining manuscript.

6. Concerning Comoros, a paper was published in 2007 describing response teams (https://nam02.safelinks.protection.outlook.com/?url=https%3A%2F%2Fwww.researchgate.net%2Fpublication%2F229983054_Needs_for_an_Integrative_Approach_of_Epidemics_The_Example_of_Cholera%2Flink%2F59e5d180a6fdcc1b1d96f21d%2Fdownload&amp;data=04%7C01%7Cpbspiegel%40jhu.edu%7Cf6902f7fc6b943fb00a908d9a2be0e1e%7C9fa4f438b1e6473b803f86f8aedf0dec%7C0%7C0%7C637719760656710690%7CUnknown%7CTWFpbGZsb3d8eyJWIjoiMC4wLjAwMDAiLCJQIjoiV2luMzIiLCJBTiI6Ik1haWwiLCJXVCI6Mn0%3D%7C3000&amp;sdata=9V%2F6iaTBZfLCAdlXRfZeIUa7e8t3eNMr6Ls7EycAJoM%3D&amp;reserved=0), and I believe it should be included in this systematic review as well.

Thank you. Since our search dates were between January 2009 and November 2019, we will not be able to include this article in the systematic review.

7. Page 14: As far as I know, no reactive OCV was implemented in Haiti; and no vaccinator was member of CATI teams in Haiti. As the sentence mix Haiti and South Sudan, it is confusing .

Thank you. We revised lines 228-229 as: “For instance, a nurse accompanied CATI teams in Haiti to administer antibiotics and vaccinators were CATI members in South Sudan to provide OCV.”

8. Concerning RDT and culture use for CATI implementation in Haiti, authors could easily complete their case study with informants already interviewed from Unicef, University of Notre Dame, or Assistance Publique Hôpitaux de Marseille .

Thank you. We added lines 241-242: “RDT was used in Haiti during 2013 and discontinued afterward because of delays to obtain results and reliability concerns.”

9. I am sorry I do not understand the source of the $1,776 monthly cost in Yemen, as the Unicef reference [13] states : « In Yemen, the average monthly cost range is US$1,500,000 – 1,875,000 for an average of 625 teams, with costs varying depending on rural and urban settings.43 This results in an average monthly cost of approximately US$2,400 for urban teams and US$3,000 for rural teams. » (p. 22 )

Thank you, we updated line 319 as: “…and $2,400 (urban) to $3,000 (rural) in Yemen.[13]”

10. Line 284-285: it is unclear to me what « antibody prophylaxis » state for. It usually refers to the use of monoclonal antibodies for post-exposure prophylaxis against an infectious diseases (such as rabies, hepatitis B, or COVID-19). Of course, this does not exist for cholera. I think authors aimed to refer to « antibiotic prophylaxis ».

Thank you, we changed line 300 to “antibiotic”.
